# Supplementary figures and images for: Oropouche orthobunyavirus in Urban Mosquitoes: Vector Competence, Coinfection, and Immune System Activation in Aedes aegypti
Source: Viruses. 2025 Mar 28;17(4):492. doi: 10.3390/v17040492 (PMC12031340; doi:10.3390/v17040492)

**A**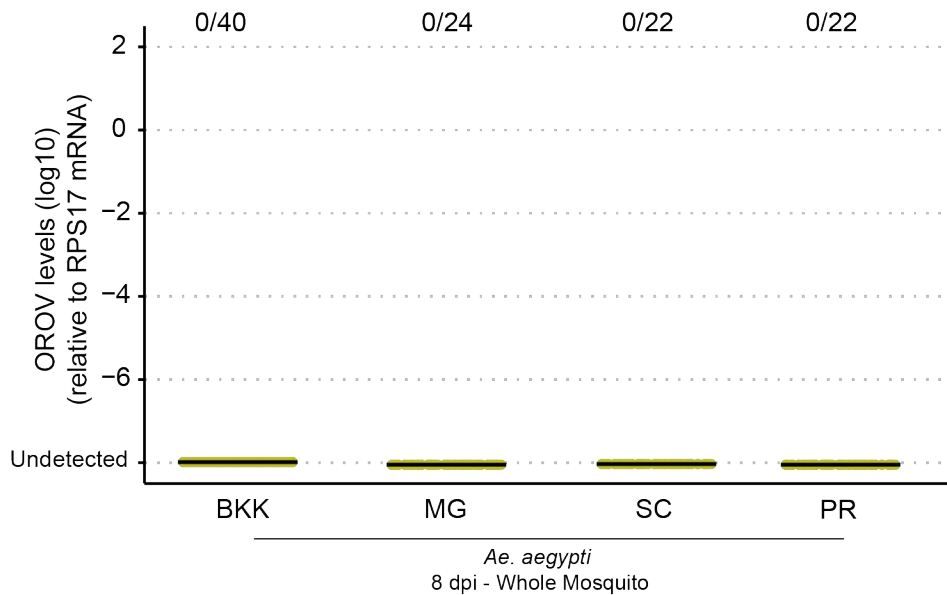**B**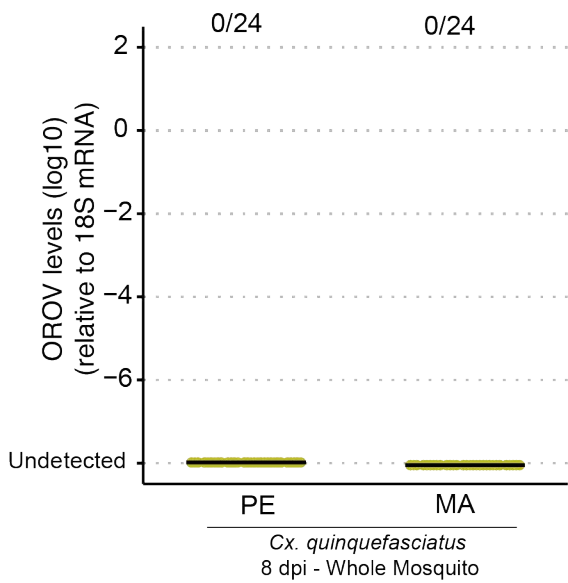

Supplement: Supplementary file 1 [file viruses-17-00492-s001.zip › Figure1S.pdf]

**A****OROV**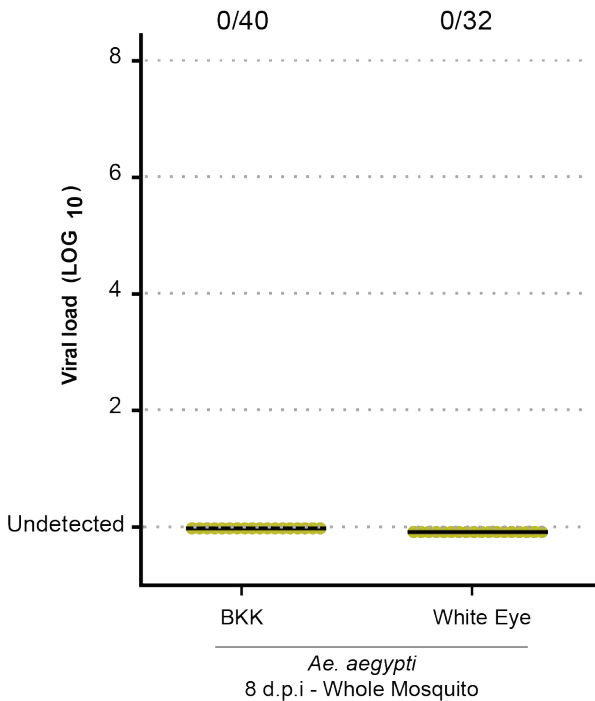**B****DENV**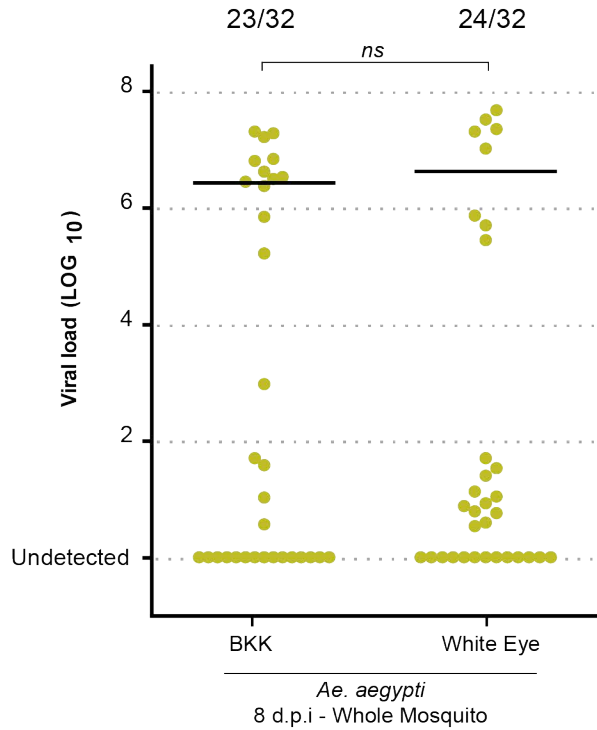

Supplement: Supplementary file 1 [file viruses-17-00492-s001.zip › Figure2S.pdf]

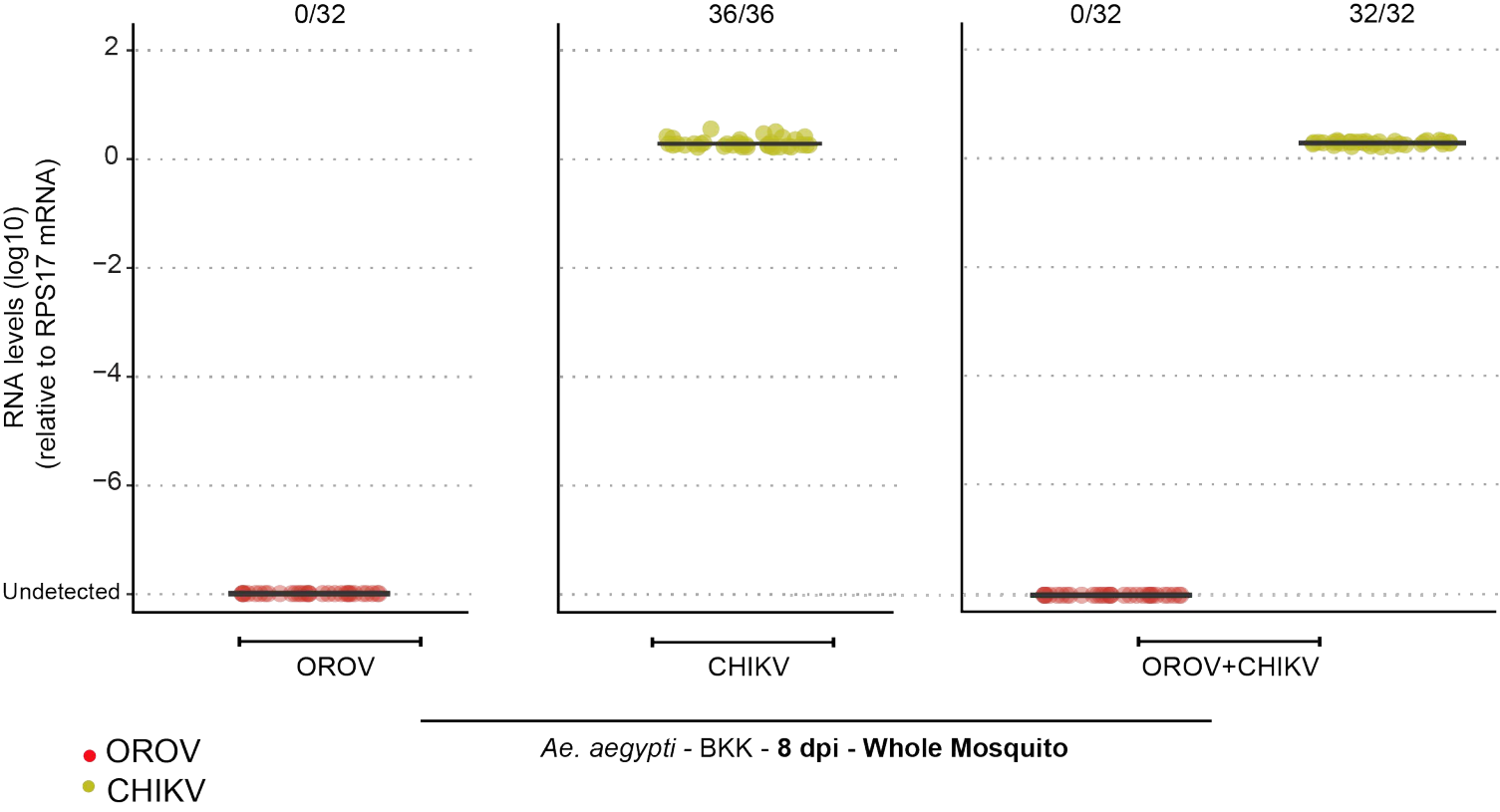

Supplement: Supplementary file 1 [file viruses-17-00492-s001.zip › Figure3S.pdf]

**A**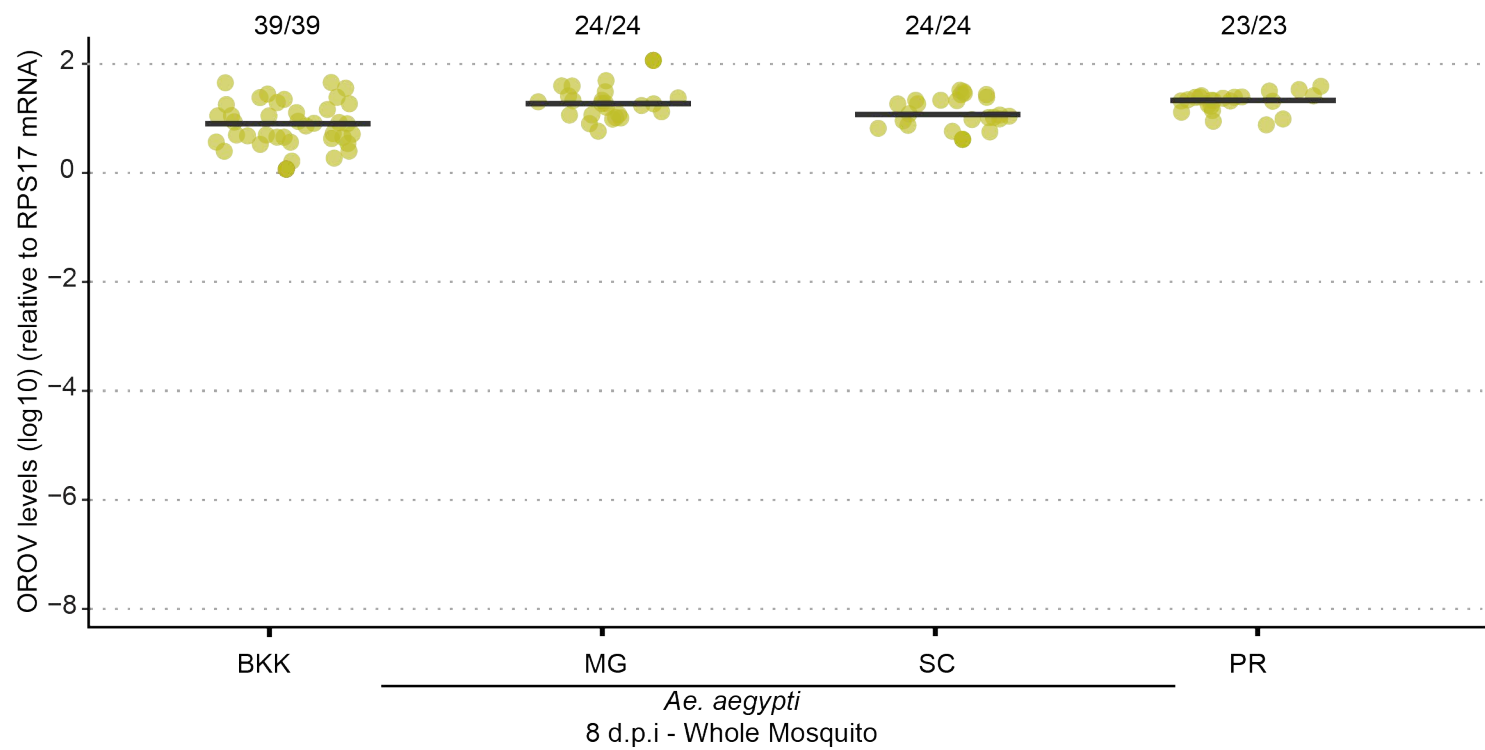**B**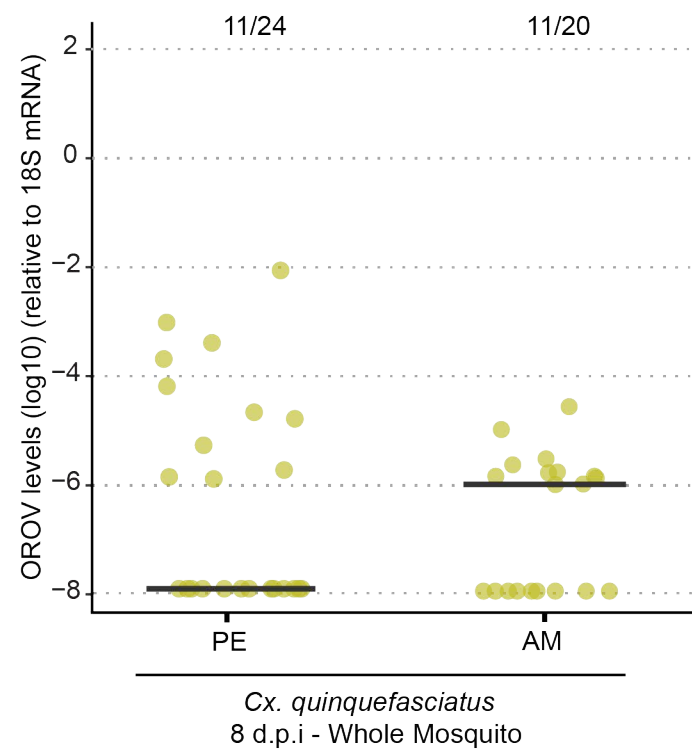

Supplement: Supplementary file 1 [file viruses-17-00492-s001.zip › Figure4S.pdf]

**A**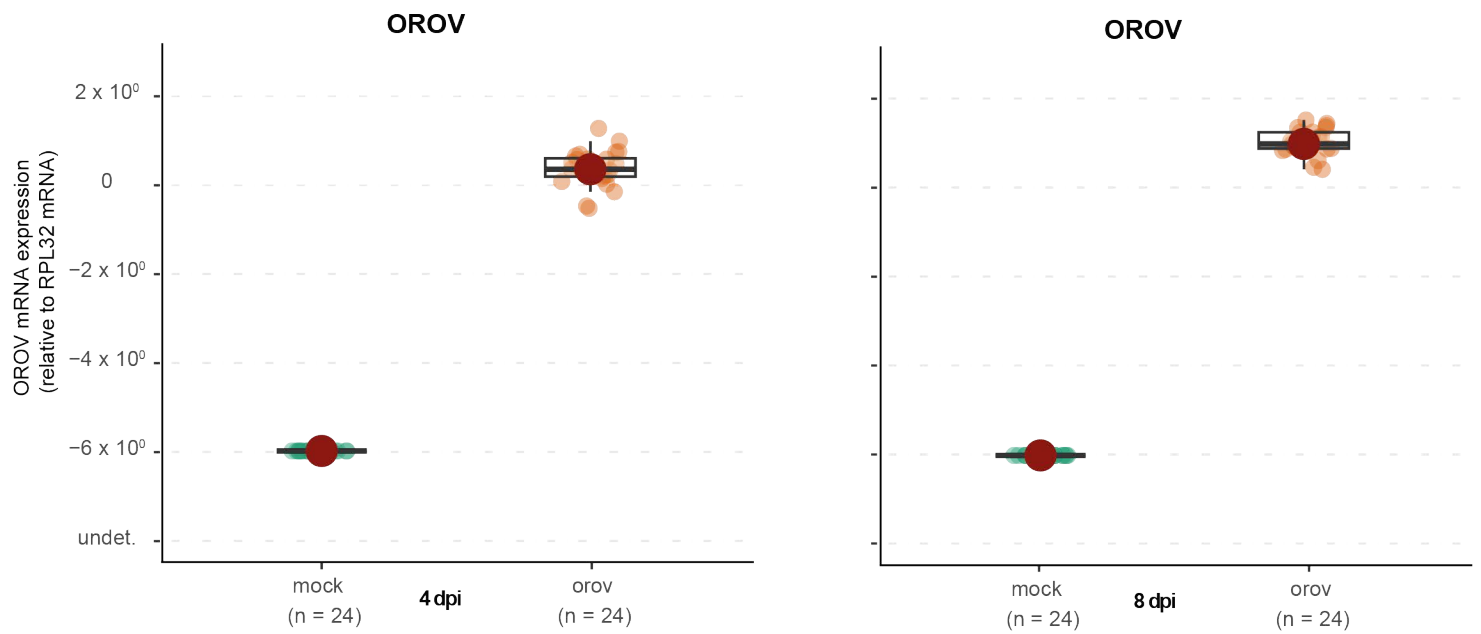**B**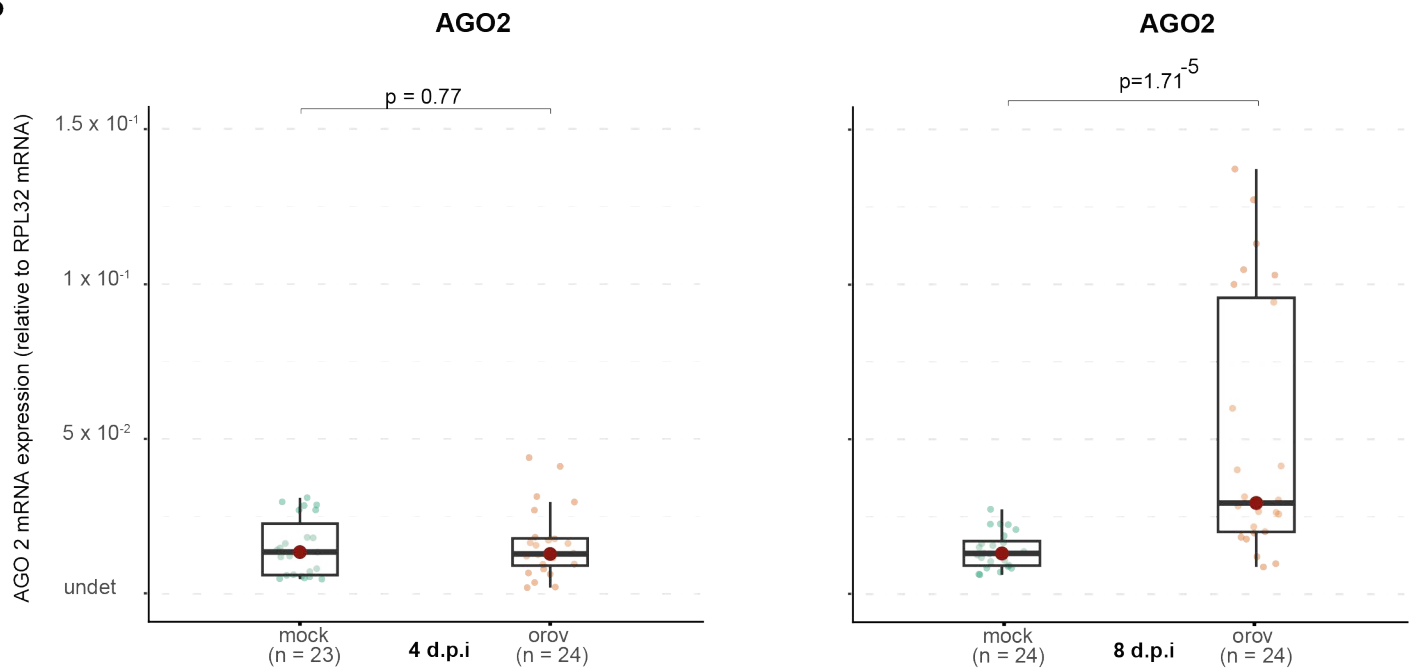**C**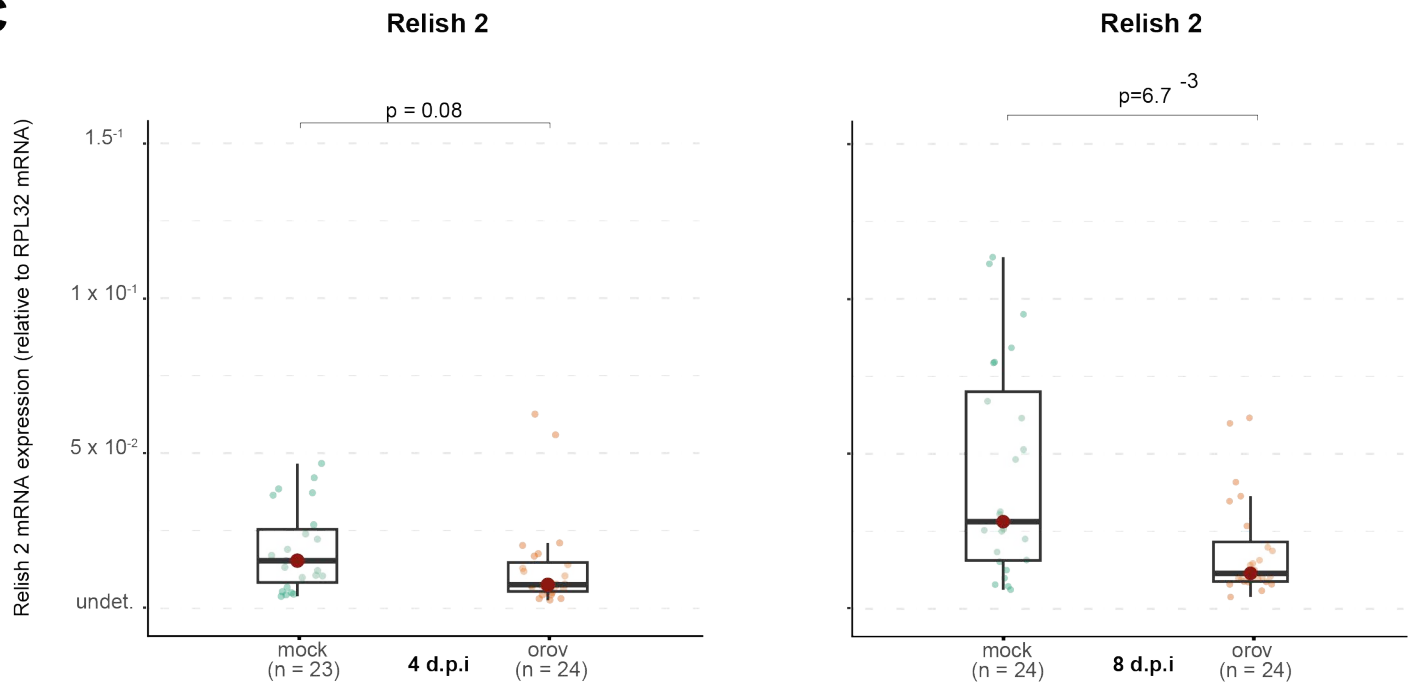

Supplement: Supplementary file 1 [file viruses-17-00492-s001.zip › Figure5.pdf]

**D**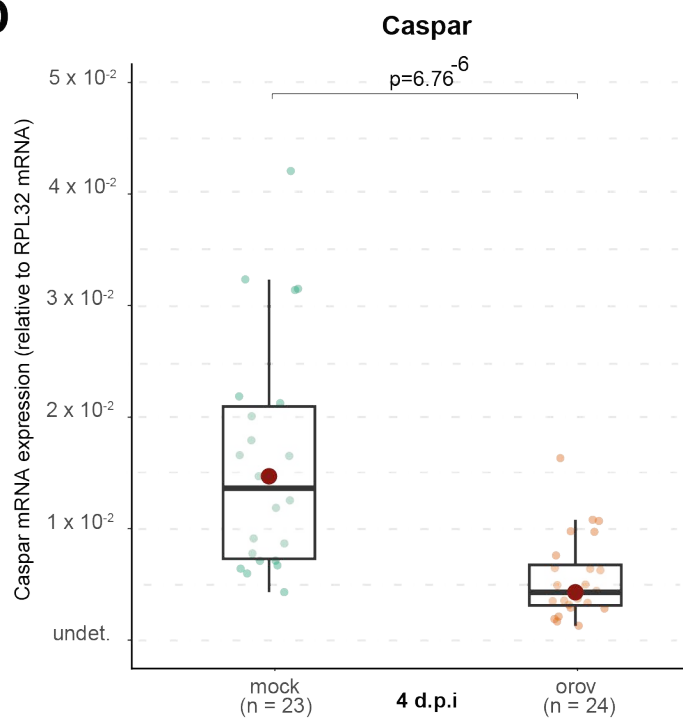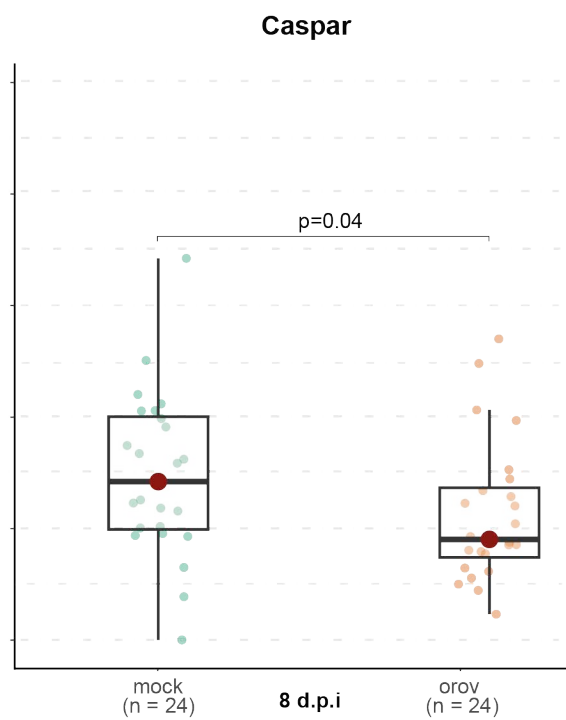**E**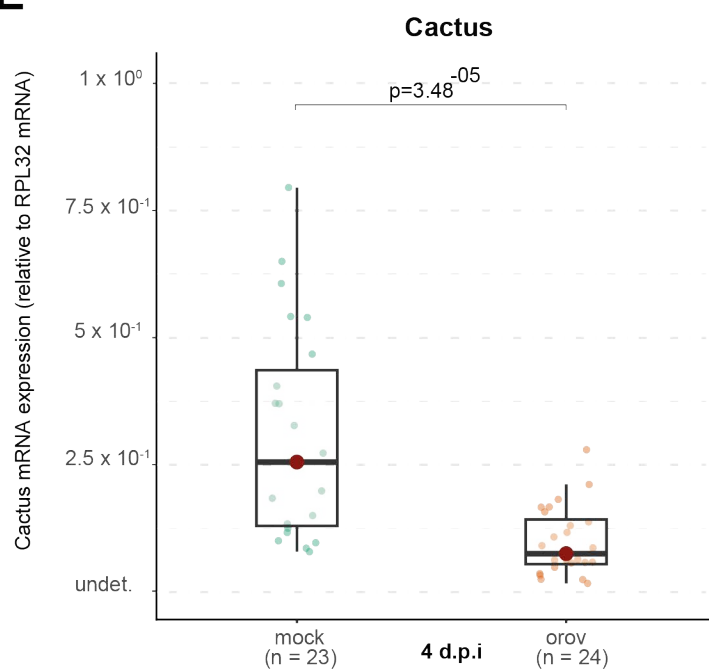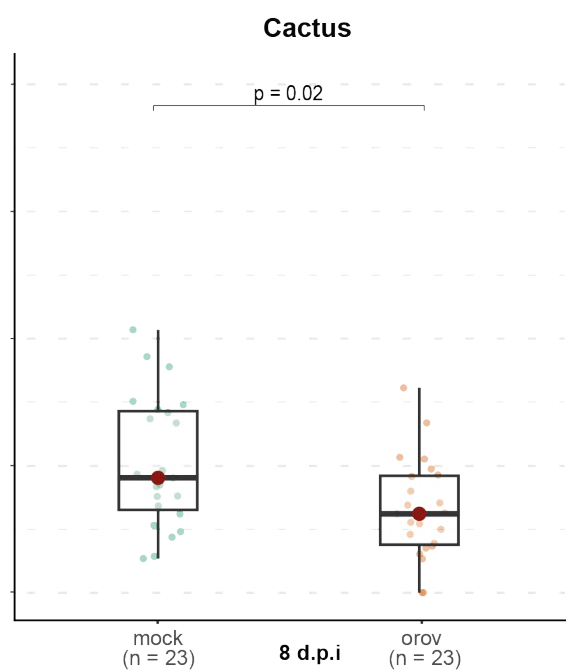**F**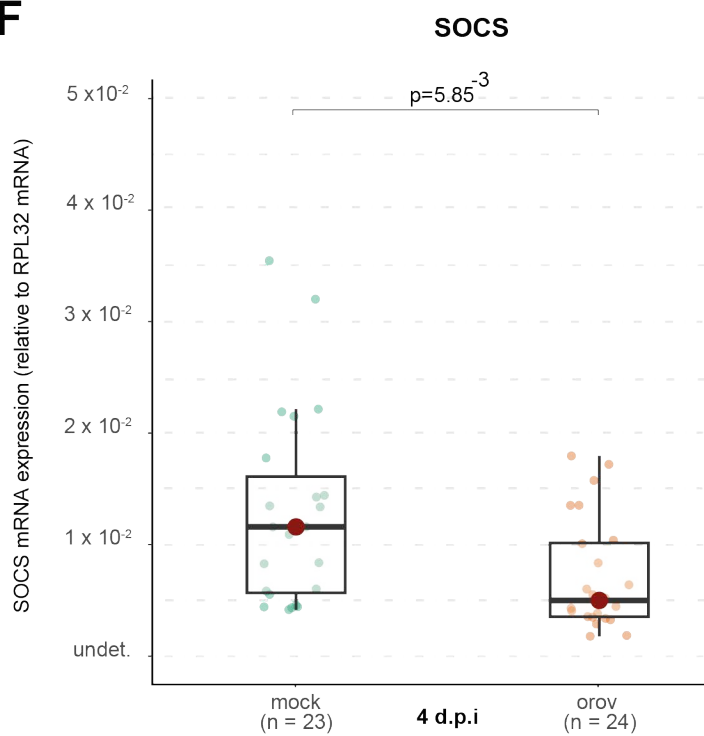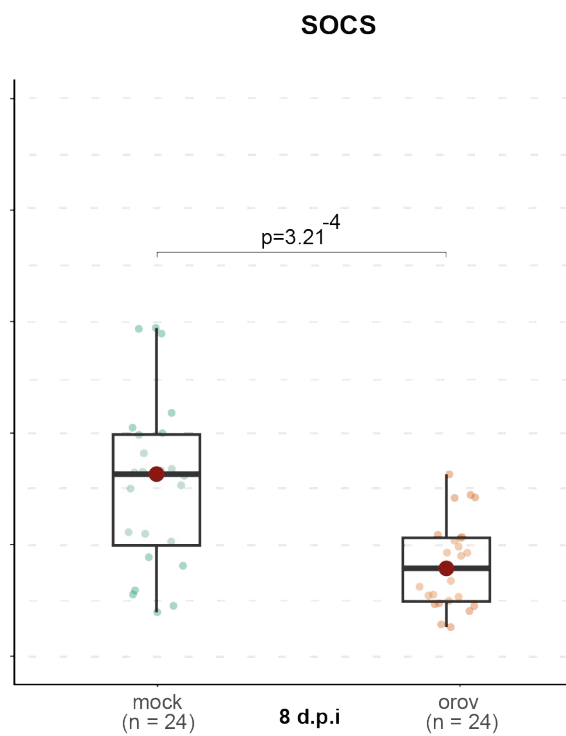

Supplement: Supplementary file 1 [file viruses-17-00492-s001.zip › Figure6.pdf]
